# Mammographic density and serum 25-hydroxyvitamin D levels

**DOI:** 10.1186/1743-7075-11-18

**Published:** 2014-04-17

**Authors:** Katherine D Crew, Julie Campbell, Diane Reynolds, Loralee Fulton, Julie D Flom, Yuyan Liao, Parisa Tehranifar, Mary Beth Terry

**Affiliations:** 1 Department of Medicine, College of Physicians and Surgeons, Columbia University, New York, NY, USA; 2 Department of Epidemiology, Mailman School of Public Health, Columbia University, New York, NY, USA; 3 The Herbert Irving Comprehensive Cancer Center, Columbia University, 161 Fort Washington Ave, 10-1072, New York, NY 10032, USA; 4 School of Nursing, Long Island University, Brooklyn Campus, Brooklyn, NY, USA; 5 Othmer Cancer Center, Long Island College Hospital, Brooklyn, NY, USA

**Keywords:** Vitamin D, 25-hydroxyvitamin D, Breast cancer, Mammographic density

## Abstract

**Background:**

Vitamin D, which influences cellular proliferation and breast tissue characteristics, has been inversely correlated with breast cancer risk. Dietary vitamin D intake has been associated with lower mammographic density (MD), a strong intermediate marker of breast cancer risk.

**Findings:**

We examined the relationship between MD and serum 25-hydroxyvitamin D [25(OH)D], an integrated measure of vitamin D status from dietary sources and sunlight exposure, in a multi-ethnic cohort of women undergoing screening mammography. We recruited women age 40–60 years without a history of breast cancer at the time of their routine screening mammogram, and conducted in-person interviews and collected blood specimens. We enrolled 195 women from 2007–2008, 120 gave blood, and 114 were evaluable, including 25% white, 41% African American, 18% African Caribbean, and 16% Hispanic. We digitized mammograms and calculated percent density, dense area, and non-dense area on cranial-caudal images. We measured serum 25(OH)D in batched, archived specimens. Median serum 25(OH)D was 22 ng/ml (range, 8–66 ng/ml). In univariable analysis, higher serum 25(OH)D was associated with white race, higher educational level, ever breast feeding, and blood draw during the summer. After adjusting for body mass index and other confounders, we found no association between serum 25(OH)D and different measures of MD. However, when stratified by season, 25(OH)D was inversely associated with dense area during July-December (p = 0.034).

**Conclusions:**

Overall, our findings suggest that circulating vitamin D, a potentially modifiable breast cancer risk factor, is not associated with MD; the seasonal effects we observed need to be replicated in larger cohorts.

## Introduction

Vitamin D has a number of anti-tumor properties, including inhibition of cell proliferation and induction of apoptosis and differentiation [1]. In observational studies of breast cancer and vitamin D status, women in the highest quantile of circulating vitamin D had a 45% reduced risk of breast cancer compared to those in the lowest quantile [2].

However, the effect of vitamin D on mammographic density (MD), one of the strongest predictors of breast cancer, remains unclear. MD refers to the relative proportions of radiolucent fat and radiodense fibroglandular tissue within the breast on mammography [3]. Serum 25-hydroxyvitamin D [25(OH)D] provides an integrated measure of vitamin D status from diet, supplements, and sunlight exposure and is considered the best indicator of vitamin D body stores [4]. We examined the association between MD and serum 25(OH)D in a cross-sectional study of racially/ethnically diverse women undergoing screening mammography.

## Methods

We enrolled 195 women age 40–60 years without a personal history of breast cancer to the New York City Multiethnic Breast Cancer Project from January 2007 to April 2008. Participants were enrolled at their routine screening mammography visits at Long Island College Hospital (LICH) in Brooklyn, New York, as previously described [5]. All participants completed in-person interviews providing information on demographics and breast cancer risk factors and consented to allow study investigators access to their mammograms; 120 (62%) participants provided a blood sample and 114 were evaluable for serum 25(OH)D measurements [5]. The study protocol was approved by the Institutional Review Boards of LICH, Long Island University and Columbia University Medical Center (CUMC).

We measured serum 25(OH)D in batched, archived specimens by Diasorin radioimmuoassay (Stillwater, MN). Interassay precision for quality controls were 14% and 18% at 15 ng/ml and 48 ng/ml, respectively. We evaluated digitized mammograms for dense area (cm^2^), non-dense area (cm^2^), and percent density (dense area divided by total breast area) on cranial-caudal images using Cumulus software, as previously described [5]. The Pearson correlation coefficients for repeated readings of a randomly selected 10% subset of mammograms were 0.99 and 0.9 for breast area and dense area, respectively.

We performed 2-sample t-tests and chi-square tests to determine whether the distribution of selected breast cancer risk factors differed by serum 25(OH)D. We used linear regression models to investigate the association of MD with serum 25(OH)D. We assessed for confounding by using the change in estimate criteria of 10% or more for estimates of the association between serum 25(OH)D and MD after adding each known potential confounding variable to the bivariable model. The multivariable models included age, race/ethnicity, education, body mass index (BMI, kg/m^2^), ever breast-feeding, and season of blood draw (January-March, April-June, July-September, October-December). We also conducted stratified analyses based upon menopausal status, BMI (≤30 vs. >30 kg/m^2^), and season (January-June vs. July-December). All statistical analyses were conducted using SAS version 9.2 (Cary, NC).

## Findings

The median serum 25(OH)D for the entire sample was 22 ng/ml (range 8–66 ng/ml). Forty-seven (41%) women had serum 25(OH)D levels in the deficient range (<20 ng/ml), 32 (28%) in the insufficient range (20–29 ng/ml), and 35 (31%) in the sufficient range (≥30 ng/ml). Baseline characteristics according to groups above and below the median serum 25(OH)D are shown in Table 1. In univariable analysis, having a serum 25(OH)D above the median was associated with white race, higher educational level, ever breast feeding, blood draw during the summer, and a trend toward lower dense area (p = 0.086).

**Table 1:** Baseline characteristics of New York city multiethnic breast cancer project (N = 114) by serum 25(OH)D levels above and below the median; 2007-2008

**Characteristic**	**Total (N = 114)**	**Serum 25(OH)D**		**P-value**^**b**^
		**≤ Median**^**a**^	**> Median**^**a**^	
Mean age at interview, years (SD)	50.0 (5.8)	49.4 (5.9)	50.7 (5.6)	0.233
Race/ethnicity, N (%)				
White	28 (25)	8 (14)	20 (36)	0.008
African American	47 (41)	32 (54)	15 (27)	
African Caribbean	21 (18)	9 (15)	12 (22)	
Hispanic/Other	18 (16)	10 (17)	8 (15)	
Menopausal status, N (%)				
Pre/perimenopausal	73 (64)	41 (69)	32 (58)	0.209
Postmenopausal	41 (36)	18 (31)	23 (42)	
Highest level of education, N (%)				
≤High school	34 (30)	22 (37)	12 (22)	0.045
Some college/associate degree	35 (31)	20 (34)	15 (27)	
≥College degree	45 (39)	17 (29)	28 (51)	
Mean body mass index, kg/m^2^ (SD)	29.2 (6.3)	30.3 (6.1)	28.1 (6.3)	0.055
First-degree family history of breast cancer, N (%)				
Yes	17 (15)	10 (17)	7 (13)	0.554
No	96 (85)	49 (83)	47 (87)	
Mean age at menarche, years (SD)	12.5 (1.8)	12.3 (1.8)	12.6 (1.8)	0.366
Mean parity, N (SD)	1.6 (1.4)	1.5 (1.5)	1.7 (1.3)	0.534
Mean age at first live birth, years (SD)	22.6 (6.8)	21.7 (6.9)	23.5 (6.7)	0.248
Ever breast fed, N (%)				
Yes	46 (40)	17 (29)	29 (53)	0.009
No	68 (60)	42 (71)	26 (47)	
Ever hormonal birth control use, N (%)				
Yes	78 (68)	43 (73)	35 (64)	0.289
No	36 (32)	16 (27)	20 (36)	
Season of blood draw, N (%)				
January-March	62 (54)	34 (58)	28 (51)	0.017
April-June	29 (25)	19 (32)	10 (18)	
July-September	19 (17)	6 (10)	13 (24)	
October-December	4 (4)	0 (0)	4 (7)	
Mean percent density, % (SD)	12.9 (11.4)	13 (11.1)	12.7 (11.8)	0.875
Mean dense area, cm^2^ (SD)	17.4 (14.7)	19.7 (16.1)	14.9 (12.8)	0.086
Mean non-dense area, cm^2^ (SD)	148 (82.3)	158 (78.2)	137 (85.9)	0.185

^a^Median 25(OH)D of 22 ng/ml.

^b^P-value based upon 2-sample t-tests for continuous variables and chi-square tests for categorical variables.

After adjusting for age, race/ethnicity, education, BMI, ever breast feeding, and season of blood draw, we found no association between 25(OH)D and the different measures of MD (Table 2), even when stratified by menopausal status and BMI. We did, however, observe a trend toward lower percent density during the late summer (July-September), when the highest levels of serum 25(OH)D were observed. Stratified analysis by season (Table 3) revealed that serum 25(OH)D was inversely associated with dense area in multivariable analysis (p = 0.034) during the months of July-December.

**Table 2:** **Multivariable linear regression estimates of percent density (%), dense area (cm**^**2**^**), and non-dense area (cm**^**2**^**), New York city multiethnic breast cancer project, 2007-2008**

**Characteristic**	**β coefficient**	**95% CI**		**P-value**
*Percent Density (%)*				
Serum 25(OH)D (ng/ml)	−0.013	−0.188	0.161	0.880
Age at mammogram (years)	−0.576	−0.921	−0.230	0.001
Race/ethnicity				
White	Reference			
African American	1.762	−3.831	7.355	0.533
African Caribbean	3.169	−3.274	9.613	0.332
Hispanic/Other	−2.094	−8.860	4.672	0.541
Education				
≤High school	−2.017	−7.157	3.123	0.438
Some college/associate degree	−3.646	−8.600	1.308	0.147
Bachelor, master, doctoral degree	Reference			
Body mass index (kg/m^2^)	−0.503	−0.873	−0.133	0.008
Ever breast feeding				
Yes	1.038	−3.015	5.091	0.612
No	Reference			
Season of blood draw				
January-March	Reference			
April-June	0.761	−3.983	5.505	0.751
July-September	−5.398	−11.259	0.462	0.071
October-December	−5.464	−16.201	5.274	0.315
*Dense Area (cm*^*2*^*)*				
Serum 25(OH)D (ng/ml)	−0.094	−0.332	0.143	0.432
Age at mammogram (years)	−0.624	−1.101	−0.146	0.011
Race/ethnicity				
White	Reference			
African American	5.421	−2.299	13.142	0.167
African Caribbean	5.838	−2.899	14.576	0.188
Hispanic/Other	−2.643	−11.985	6.700	0.576
Education				
≤High school	−0.243	−7.254	6.767	0.945
Some college/associate degree	−3.547	−10.372	3.278	0.305
Bachelor, master, doctoral degree	Reference			
Body mass index (kg/m^2^)	−0.170	−0.680	0.341	0.511
Season of blood draw				
January-March	Reference			
April-June	3.430	−3.128	9.988	0.302
July-September	−6.680	−14.763	1.403	0.104
October-December	−6.718	−21.564	8.128	0.371
*Non-Dense Area (cm*^*2*^*)*				
Serum 25(OH)D (ng/ml)	0.209	−0.823	1.242	0.688
Age at mammogram (years)	3.346	1.302	5.390	0.002
Race/ethnicity				
White	Reference			
African American	12.640	−20.455	45.735	0.533
African Caribbean	6.186	−31.943	44.315	0.748
Hispanic/Other	−2.090	−42.125	37.946	0.918
Education				
≤High school	22.854	−7.560	53.269	0.139
Some college/associate degree	21.506	−7.809	50.821	0.149
Bachelor, master, doctoral degree	Reference			
Body mass index (kg/m^2^)	7.142	4.952	9.331	<.001
Ever breast feeding				
Yes	−17.582	−41.566	6.402	0.149
No	Reference			
Season of blood draw				
January-March	Reference			
April-June	−0.074	−28.145	27.997	0.996
July-September	23.080	−11.597	57.757	0.190
October-December	8.404	−55.132	71.940	0.794

**Table 3:** **Multivariable linear regression estimates of dense area (cm**^**2**^**) stratified by season of blood draw (January-June, July-December), New York city multiethnic breast cancer project, 2007-2008**

**Characteristic**	**β coefficient**	**95% CI**		**P-value**
*January-June (N = 91)*				
Serum 25(OH)D (ng/ml)	−0.046	−0.338	0.246	0.757
Age at mammogram (years)	−0.813	−1.421	−0.205	0.009
Race/ethnicity				
White	Reference			
African American	6.014	−3.463	15.491	0.210
African Caribbean	8.406	−2.912	19.724	0.143
Hispanic/Other	−2.480	−13.841	8.882	0.665
Education				
≤High school	−1.405	−9.837	7.027	0.741
Some college/associate degree	−4.541	−13.109	4.026	0.295
Bachelor, master, doctoral degree	Reference			
Body mass index (kg/m^2^)	0.056	−0.540	0.653	0.851
*July-December (N = 23)*				
Serum 25(OH)D (ng/ml)	−0.254	−0.487	−0.022	0.034
Age at mammogram (years)	−0.272	−0.711	0.167	0.204
Race/ethnicity				
White	Reference			
African American	5.525	−1.281	12.332	0.103
African Caribbean	1.711	−5.984	9.406	0.639
Hispanic/Other	3.002	−8.678	14.682	0.588
Education				
≤High school	0.492	−6.070	7.053	0.874
Some college/associate degree	−1.411	−7.427	4.604	0.621
Bachelor, master, doctoral degree	Reference			
Body mass index (kg/m^2^)	−0.892	−1.323	−0.462	0.001

## Discussion

Overall, we observed a non-significant trend toward lower dense area in participants with higher serum 25(OH)D, which was no longer significant after adjustment for BMI and other confounders. However, during the months of July-December, we observed a significant inverse association between serum 25(OH)D and dense area, which may be a better a measure of MD in obese women.

A recent systematic review of fourteen studies examining the association between vitamin D and MD [6] included twelve cross-sectional studies [7-18] and two prospective studies [19,20]. Nine studies assessed vitamin D status by dietary and supplement intake [12-20] and five studies by circulating 25(OH)D levels [7-11]. Only four studies considered dense area as a measure of MD [7,10,11,15] and five studies included populations which were diverse by race and ethnicity [10,11,15-17].

Five out of nine studies which assessed dietary intake of vitamin D reported a significant inverse association between vitamin D and MD [8,13-15,18]. When stratified by menopausal status, much of the association was limited to premenopausal women [13-15]. In a sub-study of the Women’s Health Initiative (WHI), no association was observed between vitamin D or calcium intake and MD among postmenopausal women, however, supplemental vitamin D use was associated with lower density in younger women [17]. Of note, we did not observe an association between 25(OH)D and MD when stratified by menopausal status, however, this subgroup analysis was limited by our relatively small sample size.

More recent studies that assessed serum 25(OH)D levels in relation to MD have reported null findings [7-11]. One study found that women in the highest quartile of serum 25(OH)D had the lowest density measurements, although no significant relationship between serum 25(OH)D levels and MD was reported [7]. No association was found between circulating levels of 25(OH)D and MD among postmenopausal women in the Nurses’ Health Study. However, women in the highest tertile of MD and lowest tertile of plasma 25(OH)D had a 4-fold increased risk of breast cancer compared to women with the lowest MD and highest plasma 25(OH)D [9].

A limitation of all of these studies was that the blood collections for serum 25(OH)D and mammograms were not conducted at the same time point (with time intervals varying from 1–8 years), unlike our study in which the blood sample and mammogram were collected on the same day. Although most studies adjusted for time between blood draws and mammograms, this may not account for the seasonal variation in vitamin D status. Brisson *et al.* reported synchronized seasonal variations of MD and 25(OH)D blood levels, demonstrating that the lowest breast density was observed in early December, approximately 4 months after peak serum 25(OH)D [8]. During July-December when serum 25(OH)D are at their highest levels, we observed an inverse association between vitamin D status and dense area.

We measured serum 25(OH)D in batched archived blood samples using the well-validated Diasorin radioimmunoassay. Prior research has demonstrated that circulating 25(OH)D is very stable in serum with long-term storage [21]. There is increasing use of LS-MS technology, which allows for quantification of 25(OH)D_2_ and 25(OH)D_3_ separately. However, the clinical utility of separately measuring D2 and D3, as opposed to total 25(OH)D, is uncertain [21].

Percent density is a measurement of the dense breast tissue relative to non-dense, primarily fat tissue, and as such percent density partly accounts for differences in breast size. However, it may underestimate MD in obese women with large amounts of fat. We also assessed dense area and non-dense area, which were only evaluated in four other studies as measures of MD [7,10,11,15]. Given that the mean BMI of our study population was relatively high at 29.2 kg/m^2^, dense area may be a more accurate way of assessing breast cancer risk in these women. Obesity has been positively associated with postmenopausal breast cancer risk [22] and is also inversely related to vitamin D status. People who are overweight and obese have a higher prevalence of vitamin D deficiency compared to lean individuals due to decreased bioavailability of fat-soluble vitamin D and sequestration in adipose tissue [23].

The main limitation of our study is the relatively small sample size. Strengths of our study include the large African American sample, comprehensive examination of risk factors, adjustment for season, use of an objective measure of vitamin D exposure, collection of blood and mammograms at the same time point, and assessment of three different measures of MD. In particular, dense area is less studied and may be more relevant in a study population with a high prevalence of obesity.

In conclusion, serum 25(OH)D status, a potentially modifiable breast cancer risk factor, was not associated with MD in this cross-sectional study. However, we did observe an inverse association with dense area based upon season, which needs to be replicated in larger studies. Since a one-time measurement of serum 25(OH)D may not reflect lifetime exposure to vitamin D, future prospective studies should examine changes in vitamin D exposure over time in relation to MD and breast cancer risk, since few longitudinal studies to date have documented changes in vitamin D status with changes in MD over time [24].

## Abbreviations

BMI: Body mass index; CUMC: Columbia University Medical Center; IGF-I: Insulin-like growth factor-I; IGFBP-3: Insulin-like growth factor binding protein-3; LICH: Long Island College Hospital; MD: Mammographic density; NSABP: National surgical adjuvant breast and bowel project; WHI: Women’s health initiative; 25(OH) D: 25-hydroxyvitamin D.

## Competing interests

The authors have no potential competing interests to disclose.

## Authors’ contributions

DR participated in the design and coordination of the study and helped to draft the manuscript. JC participated in the design and coordination of the study and drafted the manuscript. JDF conducted the mammographic density measurements, participated in the design and coordination of the study and helped to draft the manuscript. KDC conceived of the study, participated in its design and coordination and drafted the manuscript. LF participated in the design and coordination of the study and helped to draft the manuscript. MBT conceived of the study, participated in its design and coordination, and drafted the manuscript. PT participated in the design and coordination of the study and helped to draft the manuscript. YL performed the statistical analysis, participated in the design and coordination of the study and helped to draft the manuscript. All authors read and approved the final manuscript.
